# Ethical considerations of research policy for personal genome analysis: the approach of the Genome Science Project in Japan

**DOI:** 10.1186/s40504-014-0004-9

**Published:** 2014-04-05

**Authors:** Jusaku Minari, Tetsuya Shirai, Kazuto Kato

**Affiliations:** Department of Biomedical Ethics and Public Policy, Graduate School of Medicine, Osaka University, 2-2 Yamadaoka, Suita, Osaka, 565-0871 Japan; Research Administration Office, Kyoto University, Kyoto, Japan; Institute for Integrated Cell-Material Sciences (iCeMS), Kyoto University, Kyoto, Japan

**Keywords:** ELSI, Personal genomics, Informed consent, E-governance

## Abstract

As evidenced by high-throughput sequencers, genomic technologies have recently undergone radical advances. These technologies enable comprehensive sequencing of personal genomes considerably more efficiently and less expensively than heretofore. These developments present a challenge to the conventional framework of biomedical ethics; under these changing circumstances, each research project has to develop a pragmatic research policy. Based on the experience with a new large-scale project—the Genome Science Project—this article presents a novel approach to conducting a specific policy for personal genome research in the Japanese context. In creating an original informed-consent form template for the project, we present a two-tiered process: making the draft of the template following an analysis of national and international policies; refining the draft template in conjunction with genome project researchers for practical application. Through practical use of the template, we have gained valuable experience in addressing challenges in the ethical review process, such as the importance of sharing details of the latest developments in genomics with members of research ethics committees. We discuss certain limitations of the conventional concept of informed consent and its governance system and suggest the potential of an alternative process using information technology.

## Introduction

The completion of the Human Genome Project (HGP) has brought about a dramatic advance in the life sciences, especially genomics (International Human Genome Sequencing Consortium 2004; Green et al. 2011). Recent genomics research has produced large quantities of personal genome data by means of next-generation sequencers and information-analysis techniques (Mardis 2011; Shendure and Aiden 2012). These technological developments have enabled international research projects to sequence personal genomes on a massive scale, such as the 1000 Genomes Project (The 1000 Genomes Project Consortium 2010) and the International Cancer Genome Consortium (ICGC) (The International Cancer Genome Consortium 2010). In these projects, researchers are required to work together cooperatively and effectively, and they then share the personal genome data internationally (Clarke et al. 2012; Joly et al. 2012). In addition, many countries have initiated various large-scale personal genome projects at the national level, including the United States, the United Kingdom, China, and Japan.

The coming of the personal genome era brings the possibility of personalized medicine based on the individual genome; however, at the same time, it raises concerns about ethical, legal, and social implications (ELSI) (Caulfield et al. 2008), which require further consideration. As the sequenced and analyzed data become ever larger and significantly more complex, research participants face greater difficulty in understanding the contents of a study and their own rights, benefits, and risks. In this regard, many reports have addressed several key topics, such as informed consent, data sharing, and returning results (McGuire et al. 2008; Kaye et al. 2010; Tabor et al. 2011). These proposals and recommendations can be informative and useful in developing a framework for a research policy in personal genome research. For practical purposes, however, each research project and organization naturally has to create concrete policies based on the scientific, social, and cultural context in which the study is being conducted.

In Japan, some national projects have been promoted in addition to such international projects as the HGP, the International HapMap Project (The International HapMap Consortium 2005), and the ICGC. Those national projects have been funded mainly by the Ministry of Education, Culture, Sports, Science and Technology (MEXT) and the Ministry of Health, Labour and Welfare (MHLW). One such longstanding project is the MEXT-funded Biobank Japan Project, which is a disease-focused biobanking project that has collected DNA, sera, and clinical data since 2003 from more than 200,000 patients with one of 47 common diseases (Nakamura 2007).

Another long-established project funded by MEXT has operated since around 1990. That project recently completed its fourth stage, having changed both its name and purpose at each stage. Initially, this project specifically addressed the progress of the HGP; gradually, it advanced along various avenues in genomic research, including plant and animal genomes and bioinformatics, while considering the relationship between genome research and society (Itoh and Kato 2005). The fifth stage of the project, named the Genome Science Project (GSP), started in 2010. The GSP aims to support various genome researchers in Japan who are funded by MEXT and selected through an annual open call; it does so by providing the services of high-throughput DNA sequencing and high-grade information technology. From 2010 to 2013, the GSP notably supported 300 individual research projects proposed by genome researchers.

**Figure 1:**
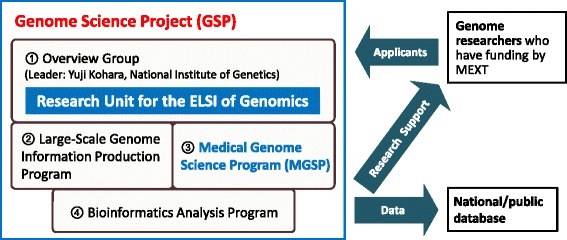
**Organizational chart of the GSP, which consists of four parts: Overview Group; Large-Scale Genome Information Production Program; Medical Genome Science Program (MGSP); and Bioinformatics Analysis Program.** Genome researchers who receive funding from the MEXT can apply to the GSP, and researchers selected through open call can receive research support. Additionally, the sequenced data obtained by the GSP are in principle entered into national and public databases.

**Figure 2:**
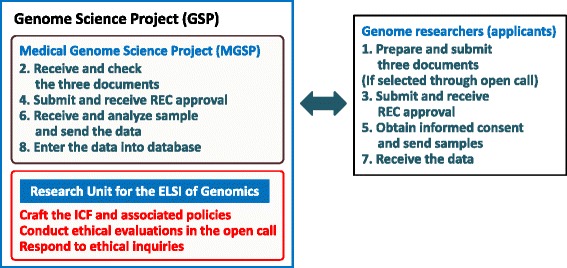
**Schematic illustration of the workflow system in the MGSP and the function of the research unit for the ELSI of genomics for the MGSP.** The applicant has to submit three documents to the MGSP: the research protocol, the informed consent form (ICF), and the MGSP application proposal. After review, both the selected researchers and associated members of the MGSP need to receive approval from the research ethics committees (RECs) of the relevant institutions. Then, the researchers have to obtain informed consent (IC) from the participants and send the specimens to the MGSP. Accordingly, the MGSP sequences and analyzes the samples; it then returns these data to the researchers, and it also enters the results into national and public databases. Here, the unit crafts the ICF template and its policy, evaluates the ethical aspects of the documents in an open-call process, and also responds to ethical inquiries from researchers.

As shown in Figure 1, the GSP consists of four subgroups. One of these is the Medical Genome Science Program (MGSP), which carries out whole human genome and exome sequencing and associated bioinformatic analysis. The MGSP also aims to create a Japanese reference genome, and it shares genome data through national public databases, such as the DNA Data Bank of Japan (Kosuge et al. 2013) and the Human Genome Variation Database (Koike et al. 2009). To address the ELSI of these actions, a specific group called the Research Unit for the ELSI of Genomics has been established within the GSP. As indicated in Figure 2, this research unit is tasked with crafting research policy and executing two ethical tasks (ethical review of documents submitted by the applicants and responding to inquiries regarding the ethical review process) in the MGSP workflow. Based on a consideration of the Japanese context, we describe in this article the approach and experience in formulating the research policy of the GSP—especially with regard to a suitable informed consent form (ICF) for personal genome research.

### Concept of informed consent and making a model ICF

The idea of informed consent has been a fundamental norm in medical ethics since the mid-twentieth century, as evident in the Nuremberg Code (US Government Printing Office 1949) and the related Declaration of Helsinki (World Medical Association 1964). Initially, informed consent was introduced as a mechanism to ensure that the participant adequately understood the scope and content of agreements, such as the purpose of the enterprise, status regarding free participation and withdrawal, and risks and benefits, with a focus on protecting individuals in clinical interventions and sample collations. Since then, the notion of informed consent has supported the right of participants in self-determination, and the scope of its application has expanded alongside the dramatic advances in biomedical and clinical research.

In human genomic research, however, the concept of informed consent differs somewhat from the original concept in certain respects. Genomic research basically uses human specimens with less invasive methodologies, and it focuses on genomic information, which affects not only the participant, but also family members and related populations (Kaye et al. 2010). Additionally, recent personal genome research has covered whole-genome information, including functionally unrevealed genome sequences, and it thus poses more unexpected risks to the participants. Moreover, although biobanking of human specimens and data sharing of personal genome data are currently conducted to facilitate further research, they potentially involve many more issues in terms of privacy and unlimited future uses of samples and data; these are the secondary, multipurpose uses by various researchers. As a result, the function of informed consent has become increasingly diversified in personal genome research.

Biomedical research in Japan is subject to soft regulation through numerous subdivided guidelines, with the exception of some areas such as drug trials (Tashiro 2010). For human genome and genetic research, in addition to the Fundamental Principles of Research on the Human Genome released in 2000 (Bioethics Committee, Council for Science and Technology 2000), national guidelines exist known as the Ethical Guidelines for Human Genome/Gene Analysis Research, which were prepared by three ministries in 2001—MEXT, MHLW, and the Ministry of Economy, Trade and Industry (MEXT, MHLW and METI 2001). Although they are not legally binding, they have strong influence over the ethical conduct of research. The 2001 guidelines describe basic principles and various issues including the method of informed consent, the return of results, and handling of samples. However, although the guidelines address general procedures, they do not detail specific instructions for each project and research institution (Slingsby et al. 2004). Especially, in the section on informed consent in the guidelines, although several subsidiary rules are listed for preparing the ICF, they cannot be readily associated with the concrete standard model of an ICF for Japan; thus, each research institute and project has to design the document independently. Furthermore, the guidelines do not deal with current conditions of personal genome research or data sharing.

In this context, we created a model ICF template for the MGSP. In the process, we had to consider major ethical issues related to personal genome research, such as data sharing and the returning of results, as detailed in other studies (Caulfield et al. 2008). Notably, the MGSP supports personal genome research at institutes across Japan, and it imposes the sharing of personal genome data on supported researchers. However, broad data sharing was previously little practiced in Japan, and it requires careful design of the ICF template, which specifically means an adequate, appropriate description of the data sharing.

In making the model ICF template, we surveyed existing informed consent documents. These were mainly collected from medical schools in Japan, as well as from international projects such as the 1000 Genomes Project, the ICGC, and the Public Population Project in Genomics. We referred to the research policies of funding agencies abroad, including those of the National Institutions of Health and Wellcome Trust. From a comparison of approximately 20 documents related to Japan, however, most did not assume a research application for the personal genome. Combining the advantages of these domestic documents and foreign documents and policies that incorporated personal genome research, we came up with a draft of a model ICF template for the MGSP that was in accordance with both Japanese government guidelines and international norms and guidelines. We believed that this approach would be beneficial in formulating a national standard that adhered to international standards.

To refine the draft of the ICF template, we held repeated discussions with executive genome researchers of the MGSP and GSP. Those individuals were representative of genome researchers in Japan and included physicians, research scientists, and bioinformaticians. The discussions were conducted over a period of 2 months in 2010 in two ways: face-to-face communication at an executive board meeting and online communication by means of an e-mail-based forum. The executive board meeting had the function of identifying controversial ethical topics in the draft of the ICF template and outlining the basic direction to be taken in that regard. The e-mail-based forum played a role in concrete considerations related to the ICF template and involved detailed, specific modifications to the document. This system worked effectively toward rapidly reaching a consensus on research policy within the MGSP: the leaders of the MGSP and GSP actively took part in the process and increasingly recognized the importance of ethical aspects in personal genome research. In addition, the cooperation with genome researchers was invaluable for incorporating pragmatic aspects of research.

The consensus regarding the research policy of the ICF template for MGSP applicants consisted of the following five key issues (Table 1): research purpose; collaboration with external institutes; data sharing; withdrawal of consent; and risk assessment and management. For a sixth key issue—returning research results and incidental findings—clear consensus could not be achieved. With regard to data sharing, there were notable difficulties in effecting an appropriate system since this was the first attempt to introduce two access levels (open-access and controlled-access levels) into informed-consent documents in Japan. In this data access scheme, controlled access, which is generally used for genotype and phenotype databases (Mailman et al. 2007; The International Cancer Genome Consortium 2010), is different from open access: controlled access requires the database user to obtain prior permission or authorization from an appropriate committee when browsing or downloading registered data. Although incorporating this two level access system into the ICF template has the potential drawback of producing a complex, confusing situation for researchers, research ethics committee (REC) members, and research participants, we adopted this approach so as to obtain support for the basic notion of data sharing through public databases and to provide information on the type and range of the shared data.

**Table 1:** **Specific requirements of the IC document to the MGSP**

**Key issues**	**Description**
**Research purpose**	• Explanation of personal genome research
	• Description of whole-genome/exome sequencing, if applicable
	• Description of general research purposes of genotype-phenotype information
**Collaborations with external institutions**	• List of names of relevant institutions and principle investigators in the MGSP
**Data sharing**	• Description of significance and purpose of data sharing
	• Two levels of access to national/public databases
**Withdrawal of consent**	• Inability of withdrawal of consent for data use after they are registered to databases
**Risk assessment and management**	• Physical interventions
	• Explanation of associated ethical, legal and social issues
	• Genetic counseling

As noted above, we were unable to reach clear consensus about the returning of results. Indeed, the disclosure of individual research results to participants is one of the most controversial areas in the ELSI of personal genomics (Knoppers et al. 2006; Wolf et al. 2008, 2012; McGuire and Lupski 2010; Levesque et al. 2011). In Japan, while there are national guidelines for human genome research, as described above, these leave decisions to the broad discretion of researchers. In our discussions with MGSP and GSP members, we learned that in Japan most personal genome studies—but not personal genome testing and analysis services—aim toward an understanding of the causes and mechanisms of diseases. Owing to the breadth of their purpose, such genome studies have little meaningful information that could be returned immediately to the participants. Furthermore, we confirmed at this point that the disclosure of research results for clinical purposes, but not incidental findings (an area still under consideration), was beyond the scope of the original research purposes. As a result, it was believed that such disclosure was not an obligation or responsibility on the part of investigators, in addition to the fact that data accuracy obtained in most basic research is generally not sufficiently high to qualify as clinical grade*.* Finally, after consideration of various research projects supported by the MGSP, we decided to leave the decision in this matter to each MGSP applicant.

In the finalized ICF template, which reflects the research policy of the MGSP, we were able to identify unique or specific characteristics related to Japanese society. First, the ICF template places a particular emphasis on accurately corresponding to developments in genome research, its purpose, and the risks for research participants. This is largely the result of historical issues in Japan related to social discrimination and stigmatization through inheritable diseases (Triendl and Gottweis 2008; Porter 2009). In some cases, such discrimination and stigmatization could lead to a negative image of genome research among the Japanese public. Second, the template comprehensively describes handling procedures of personal information and individual genetic information of research participants. This move is associated with the growing concern for privacy among the Japanese public after the enactment of the Act on the Protection of Personal Information of 2003 (Act No. 57 of 2003; Triendl and Gottweis 2008; Porter 2009; Masui 2009). This concern could lead to over-sensitivity to misuse of information on the part of research participants—even with regard to the use of information in biomedical research. Third, the template underlines the necessity to establish a robust relationship with research participants in the documentation process. This adjustment arose because several incidents occurred in Japan in around the year 2000, whereby a large number of samples were analyzed without informed consent having been obtained from research participants (Porter 2009). These circumstances could explain the persistent reluctance and skepticism on the part of participants to genome research.

In the case of applicants who wish to use existing samples, and thus do not need to employ the ICF template, we decided—after much debate—to adopt the same requirement conditions detailed above, i.e., the conditions for applicants using existing samples would be the same as those for applicants with new samples. Thus, if applicants with existing samples did not meet the requirements, they would have to obtain additional consent. Although requiring consent in this manner in the case of using existing samples places rather heavy demands on applicants, our decision resulted from a consideration of the participants: personal genome research and associated data sharing potentially have unexpected risks owing to the unfocused nature of whole-genome and exome sequencing and reidentifiability from data sharing (Rodriguez et al. 2013).

### Challenges facing the use of the ICF template and its prospects in Japan

The greatest challenge for the implementation of our project was whether the ICF template would be acceptable to the RECs of the institutes across Japan with which MGSP applicants are associated. Even if a model ICF could be created for research participants, it would be practically meaningless without approval by the RECs under the current ethical framework. As reported in previous papers, different RECs may evaluate the same protocol variously and apply the regulations in a different way (Silverman et al. 2001; Silberman and Kahn 2011). In Japan, while the governmental guidelines provide discretion and responsibility to RECs, they do not necessarily provide procedural details regarding the evaluation (Slingsby et al. 2004; Porter 2009). In addition, unlike other countries, there are no widely-used accreditation programs or centralized research ethics committee to review multicenter studies (Burman et al. 2001; Emanuel et al. 2004; Coleman and Bouësseau 2008). These issues make the outcome of the ethical review even more uncertain than those in countries with such programs and systems. Indeed, we found that several investigators supported by the MGSP did encounter some difficulties in obtaining REC approval. To address this problem, we implemented the following two measures.

One was that we developed requirement lists for the research proposal and for the informed consent document to be given to MGSP applicants. The lists detailed the information that applicants had to enter in the application documents. The need for the list for the research proposal arose from the experience that in such proposals to REC members, MGSP-supported researchers often failed to provide sufficient information about the GSP and MGSP—especially concerning their purpose and significance and the concrete details of personal genome analysis and data sharing. The need for the list regarding informed consent developed because supported researchers often mistakenly incorporated the research policy of the MGSP into the informed consent document of their institution—not the ICF template provided by the MGSP. These two requirement lists served as a practical and powerful means of reliably informing researchers of the minimum requirements for those documents and then appropriately providing the necessary information to REC members.

The second measure was that we held a workshop regarding ethical review of personal genome research (August 2012 in Tokyo). Even if researchers support the use of the research proposal and informed consent documents containing the research policy of the MGSP, we cannot necessarily guarantee REC approval in all cases. Thus, to further facilitate the ethical review process, we requested participation in the workshop by REC members and support staff of all research institutions to which the supported researchers belonged. In total, 76 people from 30 institutes participated in the workshop, during which we explained the current situation of personal genome research and data sharing in addition to the activities of the GSP and MGSP. Through this experience, we found that such workshops are useful in facilitating the sharing of current information and basic rules for the ethical review of personal genome research; regular workshops can also be used to coordinate and improve the quality of ethical review of multicenter studies.

We made several important findings with this practical use of the ICF template. The first is the necessity to rapidly share knowledge related to the latest genome research among professionals, such as researchers and REC members. The second is the necessity to foster a robust collaboration between professionals from different fields for promoting large-scale projects. In this regard, we found that if relevant professionals in Japan are aware of the challenges related to such projects, they are often more willing to collaborate. This situation may reflect the characteristics of Japanese culture; thus, it may be a desirable strategy to effect such collaboration to facilitate ethically sound research.

The most important finding for us regarding the practical use of the ICF template was the necessity to review the concept of conventional informed consent and the ethical governance system. For the governance of genome research using human specimens, the “one researcher, one project, one jurisdiction” model, whose origins are in the Nuremberg Code, has been widely adopted (Kaye 2011). With this model, informed consent is on a one-time basis and mostly front-loaded (Gostin and Hodge 1999). Accordingly, the system of traditional informed consent and RECs faces greater challenges in personal genome research. In particular, there are difficulties with the prior one-time consent approach being able to fully inform research participants of broad research plans because “the more general the consent is, the less informed it becomes” (Arnason 2004). With this project in Japan, we learned the limitation of incorporating ethical considerations in prior one-time consent because it is difficult to anticipate the future scope of research, including data sharing and the risks and benefits of whole-genome and exome sequencing. Several reports have suggested the necessity of additional or alternative procedures without relying solely on the conventional informed consent procedure (Caulfield et al. 2008; Lunshof et al. 2008; Watanabe et al. 2011; Kaye 2011).

The introduction of digitalization to the system—e-governance—is attracting attention as a means of establishing a strong relationship among stakeholders. Information technology (IT)-based consent, sometimes called dynamic consent (McGuire and Beskow 2010; Kaye et al. 2012), allows more continuous, flexible, and interactive implementation than the conventional one-time consent. This IT approach regards participants as a kind of partner, and through a Web-based infrastructure it provides them with multiple opportunities to make decisions regarding research participation. The IT infrastructure could also provide an effective supplementary tool for informed consent: it can be used to create an online community for continuous connection among researchers, bioresources, and research participants (Cambon-Thomsen et al. 2011; Fenner et al. 2011). The online system for returning results called My46 (Tabor 2012) allows participants to control the information disclosed. Although the e-governance approach still faces several technical, financial, and social issues in terms of its feasibility and public acceptability, it will undoubtedly play a significant role in respecting the flexible autonomy of research participants and lightening the future management load of researchers and REC members.

Finally, we have described in this article personal genome research policy, but not clinical practice. Recently, many major institutions have already started using whole-genome and exome sequencing in the clinical setting (Worthey et al. 2011; Bainbridge et al. 2011; Gonzaga-Jauregui et al. 2012). As a result, the discussion of the ELSI associated with clinical practice is becoming more heated (Biesecker et al. 2012). In Japan, there are some such activities in clinical application, albeit on a much smaller scale. Our experience with research policy may share a number of similarities with the situation in clinical practice, and our findings may serve at least as a starting point for policy-making activities in the clinical setting.

## Conclusion

We have presented a research policy and approach for the GSP. We reconsidered the nature of informed consent, developed an ICF template, and aimed to facilitate an understanding of the project’s policy among researchers and REC members. Formulating the ICF template taught us the difficulty in setting a specific policy regarding data sharing and returning results, and we noted the specific ethical characteristics of informed consent in Japan. With the practical use of the template, we found that sharing the latest knowledge about genome research and collaboration among various professionals were important in promoting a large-scale project that involves many research institutes. Furthermore, our experience provided an opportunity to reconsider the nature of one-time consent and the research governance system. To solve the attendant problems without resorting to the consent approach, it will be necessary to reevaluate the conventional governance system. Among several possibilities, the use of an IT-based governance system would appear to be effective in enabling dynamic communication with research participants. As an increasing number of IT-based methods are developed and become adopted, it will be possible to establish a well-balanced research governance system that can incorporate both participants’ trust and research advancement.
